# The Effect of Tranexamic Acid With or Without Tourniquet on Blood Loss in Total Knee Arthroplasty at a Community Hospital

**DOI:** 10.7759/cureus.54835

**Published:** 2024-02-24

**Authors:** Elise N Ketelaar, Michael Wagner, Adam Lorenzo, Robert Comrie, Carolina Restini, Grace D Brannan, Roger Corvasce, Saad Mohammad

**Affiliations:** 1 Orthopedic Surgery, Beaumont Health, Farmington Hills, USA; 2 Orthopedic Surgery, McLaren Macomb Hospital, Mount Clemens, USA; 3 Anesthesiology, University of Buffalo, Buffalo, USA; 4 Pharmacology, Michigan State University College of Osteopathic Medicine, Macomb, USA; 5 Orthopedic Surgery, Mclaren Macomb Hospital, Mount Clemens, USA

**Keywords:** orthopedic surgery, joint replacement, hemoglobin, tourniquet, blood loss, total knee arthroplasty, tranexamic acid

## Abstract

Tourniquets have long been used in total knee arthroplasty due to the theoretical improvement of bleeding control, integration of cement-bone interface, visibility, and efficiency of the overall surgery. However, this has become increasingly disputed. Comparative studies in total knee arthroplasty employing chemical prophylaxis, i.e., tranexamic acid, have been conducted. This retrospective cohort study evaluated the effect of tranexamic with or without a tourniquet on mean blood loss, hemoglobin, and length of stay in total knee arthroplasty patients. A total of 153 patients’ records met the inclusion criteria, 95 patients (62%) were in the tranexamic acid-only group while 58 patients (38%) were in the tranexamic acid plus tourniquet group. Based on mean blood loss in mL (827.5 without vs. 810.1 with the tourniquet, p=0.805), hemoglobin counts in g/dL (12.6 without vs. 12.5 with the tourniquet, p=0.598), and length of stay in days (1.0 days without vs. 1.1 with the tourniquet, p=0.204), there was no statistical difference between the tranexamic alone vs. tranexamic plus tourniquet groups. There were no statistical differences in the mean BMI between groups (32.3 without vs. 32.4 with tourniquets, p=0.901). The patient population had more women (64.1%) than men (35.9%) (p=0.001), but no significant difference in gender based on tourniquet use (p=0.521). The tourniquet group averaged three years younger than the tranexamic alone group (age mean 68.2 without vs 65.3 with tranexamic, p=0.029). This study found no identifiable difference in the three observed variables, indicating that tourniquet provides limited to no additional benefit in reducing blood loss over tranexamic alone in total knee arthroplasty, while tranexamic alone has no deleterious decrease in mean hemoglobin or increase in length of stay.

## Introduction

Since the procedure was first implemented, pneumatic tourniquets have been used in total knee arthroplasty (TKA) surgery. Their use was expected to reduce blood loss, optimize visualization of the operative field, increase operative efficiency, and enhance cement integration [1]. Recently, the implementation of quality improvement initiatives and intraoperative tranexamic acid (TXA) resulted in a significant decrease in total blood loss and unnecessary transfusions [2]. As a result, the necessity of tourniquet use has recently been questioned, with recent studies stating that its use results in exacerbation of postoperative pain, extended hospital stay, reduction in postoperative function and range of motion, and no significant reduction of postoperative blood loss when compared to tourniquet-less surgery [3,4]. In addition, the importance of a tourniquet to create a bloodless environment for maximal cement integration into bone has been questioned with new evidence showing good cement penetration without the use of a tourniquet [5].

The evidence supporting tourniquet use remains controversial in the literature. Recent literature demonstrates that tourniquet groups may experience significantly less intraoperative blood loss and operation time; however, they may not have a significantly reduced rate of transfusion, or deep vein thrombosis (DVT) compared to tourniquet-less groups [6]. Recent literature has demonstrated that using a tourniquet may lead to an increased risk of several serious complications, including DVT, pulmonary embolism, wound infection, and the need for reoperation [4]. Additionally, these patients frequently experience greater pain in the immediate postoperative period, with some patients consuming more analgesics in response to this pain [4,7].

In recent years, the use of TXA in orthopedic surgery for reducing blood loss has been growing and is especially important in hip and knee arthroplasty, and spinal surgery [8]. Multiple studies have outlined the effectiveness of TXA in orthopedic surgery to reduce blood loss and transfusion rates without increasing the risk of thromboembolism [8].

TXA is a synthetic lysine-analog antifibrinolytic drug that competitively inhibits the activation of plasminogen to plasmin, effectively inhibiting clot degradation by plasmin [8]. TXA has traditionally been used for bleeding disorders such as menorrhagia, as well as for reduction of perioperative bleeding and transfusion in cardiac and non-cardiac surgery [8,9]. Although there is robust data regarding TXA in orthopedic surgery, little literature is available directly comparing the use of TXA with and without tourniquet in TKA, and no guidelines have been developed.

To our knowledge, only two studies within the United States directly compare the effectiveness of TXA with and TXA without tourniquet in TKA. Both studies were performed at an academic health center. Only one of these studies evaluated their effect on hemoglobin and blood loss [10,11]. One metanalysis is available, which outlines randomized controlled trials (RCTs) performed mainly in Europe and China comparing TXA vs. tourniquet in TKA [12]. Only one study was from the US [11]. These studies have mixed conclusions regarding the effects on blood loss. Therefore, it has been recommended that more studies are needed on the topic. As institutions strive for safe and cost-effective delivery of joint arthroplasty, there is a fundamental need to investigate the efficacy and safety of tourniquets during arthroplasty and to determine alternative methods for preventing blood loss during TKA procedures. This study aimed to investigate the use of TXA with or without a tourniquet and its effect on mean hemoglobin, blood loss, and post-procedure length of stay (LOS) in TKA patients.

## Materials and methods

Study design, level of evidence, and data collection

This is a retrospective cohort study (evidence level three) approved by the McLaren Healthcare System IRB (Protocol ID: 2019-00066). We accessed the records of McLaren Macomb Hospital patients who underwent primary TKA from September 2016 to March 2019. Patient records were accessed via the ICD-10 code corresponding to TKA. In some patients, a tourniquet and TXA were used. For others, TXA was used without a tourniquet.

Patients whose records were reviewed had primary TKA, not requiring advanced implants. In our study, a combination of mechanical and robotic-assisted devices was used to perform the procedures under the supervision of a fellowship-trained total joint orthopedic surgeon and a general orthopedic surgeon.

All patients received 2,000 mg of TXA using the hospital’s standard protocol of 1,000 mg of TXA administered intravenously immediately prior to skin incision, with an additional 1,000 mg at wound closure.

Pre-operative or initial hemoglobin (Ho) values were obtained from standard medical clearance labs within two weeks prior to the operative day, and on the postoperative day, one hemoglobin was drawn to determine final hemoglobin (HF). Mean hemoglobin (HbAV) was then determined by averaging pre-operative or initial hemoglobin (Ho) and final or postoperative hemoglobin (HF) and dividing by two. Patients were excluded if they did not have an available hemoglobin drawn within two weeks prior to the operative day. Blood loss was estimated based on Gross’s formula [13]. EBV was determined using the “Estimated Blood Volume as a Function of Body Habitus” [13]. Total blood loss was then calculated by multiplying EBV by changes in hemoglobin according to the formula [13]. No minimum allowable hemoglobin or hematocrit was used.

An adductor canal block was used pre-operatively, along with standard general anesthesia for all patients. All elective TKA procedures in our institution receive intra-operative liposomal Bupivacaine (Exparel) injection as a local anesthetic. A standard midline incision with a medial parapatellar approach was used in all patients. Intra-medullary alignment jigs and appropriately sized conventional femoral and tibial cutting guides were used in non-robotic cases. Press fit vs. cemented implants were determined intra-operatively and varied case by case, depending on the bone quality. Patellar resurfacing was done in the majority of our patients; however, this was also determined intra-operatively. No drains were used post-operatively.

No patients were on blood thinners within 48-72 hours prior to surgery. In our institution, all TKA patients receive our standard post-operative pain protocol (which includes Oxycodone PRN, Tylenol Q6, and Toradol Q6). Primary DVT prophylaxis involves a combination of a pharmacological agent, Aspirin 81 mg BID, and mechanical, sequential compression devices.

Inclusion/exclusion criteria

All patients over the age of 18 who underwent primary TKA not requiring advanced implants at our level II community teaching hospital from September 2016 to March 2019 were included in the study except when they met the exclusion criteria.

Patients were excluded from the study based on the following criteria: history of pulmonary embolism, history of DVT, history of myocardial infarction or stroke within six months prior to surgery, history of peripheral vascular disease, history of renal dysfunction (glomerular filtration rate < 30 mL/min/1.73 m^2^), bilateral TKA, rheumatoid arthritis, or body mass index >40. Patients were also excluded if they did not have pre-operative hemoglobin labs drawn within two weeks of the operative day. Patients who required constrained or hinged implants were excluded.

Outcomes and statistical analyses

Descriptive statistics, including frequency and percentages, were generated. An independent t-test was performed on continuous variables and a Chi-square test was performed on categorical variables. Blood loss was the primary variable evaluated. The secondary variables were hemoglobin and LOS. IBM SPSS Statistics for Windows, Version 25.0 (IBM Corp., Armonk, NY) was used. Statistical significance was set at a p-value <0.05.

## Results

A total of 153 records of patients met the inclusion criteria. Ninety-five patients (62%) were included in the tranexamic acid-only group, while 58 (38%) patients were included in the tranexamic acid plus tourniquet group. When comparing BMI based on the tourniquet group, there was no statistically significant difference (p=0.901) between the two patient populations. However, patients with tourniquet use averaged three years younger than those with TXA (p=0.029) (Table 1).

**Table 1:** BMI and age values by tourniquet use. Discrepancies between total number of patients in the tranexamic acid without tourniquet group (95) and the BMI of patients with tranexamic acid without tourniquet (83) are due to missing data during the data collection period, therefore these patients were not included in calculations for mean BMI.

	Tourniquet Use	N	Mean	Std. Deviation	P-value
BMI [kg/m^2^]	without	83	32.3	6.37	0.901
	with	58	32.4	5.71	
Age [years]	without	95	68.2	7.62	0.029
	with	58	65.3	8.44	

Overall, in this population of TKA patients, there were significantly more women (64.1%) than men (35.9%) (p=0.001). However, there was no significant difference (p=0.521) in male and female proportions based on tourniquet use (Table 2).

**Table 2:** Gender distribution by tourniquet use.

Tourniquet	Gender			Total	P-value
	Male	Female			
Without	36 (37.9%)	59 (62.1%)	95 (100.0%)		
With	19 (32.8%)	39 (67.2%)	58 (100.0%)		
Total	55 (35.9%)	98 (64.1%)	153 (100.0%)		0.521

There was no statistical difference between the tourniquet groups based on mean blood loss (p=0.805), hemoglobin (p=0.598), and LOS in days (p=0.204) (Table 3).

**Table 3:** Mean blood loss, hemoglobin, and length of stay (LOS) in days by tourniquet group. Discrepancies between total number of patients in the tranexamic acid without tourniquet group (95) and the blood loss and length of stay of patients with tranexamic acid without tourniquet (89, 93 respectively) are due to missing data during the data collection period, therefore these patients were not included in calculations for mean blood loss and length of stay.

	Tourniquet Use	N	Mean	Std. Deviation	P-value
Blood Loss (mL)	No	89	827.5	485.8	0.805
	Yes	52	810.1	343.5	
Hemoglobin count(g/dL)	No	95	12.6	1.2	0.598
	Yes	55	12.5	1.3	
LOS (days)	No	93	1.0	0.2	0.204
	Yes	55	1.1	0.4	

## Discussion

Our study demonstrated no significant difference in mean blood loss, hemoglobin, and LOS between TKA with TXA plus tourniquet vs. TKA with TXA alone. These findings suggest that the tourniquet offers no additional benefit in terms of reducing blood loss in TKA in this population.

Intraoperative blood loss is routinely calculated by measuring the blood in the suction canister as well as operative towels; however, no agreed-upon gold standard protocol for determining blood loss exists today [14]. Rather than direct measurement, we opted for estimation via Gross’s formula. This formula accounts for pre-transfusion maintenance of intravascular volume using crystalloids, which can create inconsistencies in blood-loss estimations by gradually decreasing hematocrit [13]. Gross’s formula has been used in many published orthopedic studies in various sub-specialties and topics for the estimation of total blood loss [15]. Although this approach does not directly quantify the actual blood volume lost, it reduces the bias that could occur when estimating blood volume lost.

Current literature indicates that tourniquet use leads to increased postoperative pain and opioid consumption following TKA [7]. Therefore, the use of TXA alone could be an effective method to achieve adequate blood loss control while minimizing post-operative discomfort associated with tourniquet use and subsequently decreasing the risk of complications such as DVT, wound infection, and delayed functional recovery secondary to tissue ischemia from the tourniquet [1,3,4].

To our knowledge, our study is the first from a US community hospital perspective. There were two other studies based in the United States that directly compared the use of tourniquets combined with TXA treatment vs. TXA alone and their effects on blood loss. Both were from academic health centers [10,11]. In 2017, Schnettler and colleagues [10] found that the use of a tourniquet led to a significantly higher volume of blood loss when total blood loss was accounted for. The authors indicate that this finding may result from fibrinolysis activation following the tourniquet's release [10]. A multicenter RCT published in 2021 by Zak et al. evaluated postoperative opioid consumption in TKA with tourniquet vs. no tourniquet plus TXA as a primary goal [11]. The authors reported no significant difference in LOS between the two groups; however, unlike our study, Zak et al. did not evaluate hemoglobin count or blood loss [11].

In a study from the United Kingdom, Patel [16] found that the unassisted tourniquet group had a greater mean postoperative hemoglobin drop than the tourniquet-assisted group; however, only the unassisted tourniquet group evaluated use with TXA, and no comparison was made between TXA combined with a tourniquet [16].

One meta-analysis compared the effectiveness of tourniquets versus TXA in total knee replacement to date. Sun et al. have recently identified 14 relevant RCTs correlating the use of TXA with vs. without a tourniquet [12]. Among all the RCTs reported by the authors, seven reported blood loss to be similar between the two groups [12]. In addition, four RCTs investigated hemoglobin count, showing a comparable reduction in hemoglobin between the two groups [12]. Furthermore, nine RCTs demonstrated similar length of stay results in both groups [12]. Of note, none of the RCTs referenced in this meta-analysis had a primary goal of comparing blood loss, reduction in hemoglobin, or length of stay using a tourniquet vs. TXA alone for TKA. Additionally, only one trial included in this meta-analysis, Zak et al., involved patients in the United States [11]. The authors concluded that additional adequately powered studies were required to validate their findings [11].

In this study, we demonstrate the effectiveness of TXA for tourniquet-less TKA within a small suburban teaching hospital based in the Midwest United States. This study is one of only two available studies within the United States that directly compares TXA when combined with tourniquet vs. TXA alone in TKA, as mentioned above. However, as we have discussed, these studies have shown differing results regarding the effects on hemoglobin postoperatively.

Demographics and patient populations are of utmost importance when standardizing management and creating treatment guidelines. To prove the efficacy and applicability of treatment, it is imperative to demonstrate that the specific procedure in question is independent of demographic and biological variability. In essence, the same procedure must produce the same outcome within various settings. European and Chinese populations have differing phenotypes, and genetic polymorphisms than the patient population within the United States. These factors may affect dose responses when using pharmacologic agents such as TXA [17]. For example, homozygous (G5/G5) polymorphisms of the plasminogen activator-1 (PAI-1) gene have been linked to lower levels of PAI-1 [17]. In patients who underwent cardiac surgery, lower levels of PAI-1 cause a greater bleeding tendency as well as a more pronounced response to TXA [17]. Interestingly, PAI-1 gene polymorphisms are linked to variations in ethnicities. In particular, PAI-1 4G/5G polymorphism has been linked to an increased risk of venous thromboembolism in Asian populations [18]. This is an isolated example of polymorphism based on ethnicity/location factors that have been found to influence the effects of tranexamic acid. Consequences of other polymorphisms affecting TXA metabolism and effectiveness remain to be determined.

Our data add to the available articles supporting evidence for the safety and effectiveness of TXA in tourniquet-less TKA while providing evidence that suggests its success is independent of the patient population, pharmacogenetics, and geographic location.

Limitations

There are several limitations of this study. First, the authors did not separately evaluate intraoperative, postoperative, or hidden blood loss parameters. This information is additionally beneficial when comparing tourniquet with TXA vs. TXA alone as it gives a better understanding of which modality allows for visualization of the operating field, and how they affect blood loss during the postoperative period.

Second, the authors did not assess complications such as soft tissue damage, infection, thromboembolism, or delayed wound healing. Further studies are needed to demonstrate complication rates between the current standard of care versus the proposed alternative; however, much literature is already available regarding this topic.

It would also be beneficial to observe secondary measures such as post-operative range of motion and postoperative pain scores to demonstrate the benefits or risks between groups in the postoperative period, however, these variables were not a primary objective of this study.

In addition, the authors did not evaluate differences in the observed parameters based on the surgeon or type of operation. For example, standard vs. robotic-assisted surgeries, as well as the surgeon's level of expertise, can all affect blood loss due to the length of surgery as well as the use of intra-medullary jigs. However, recent studies have shown differing findings when comparing blood loss in conventional TKA vs. robotic-assisted TKA [19,20]. Additional studies should evaluate differences in blood loss and mean hemoglobin when using TXA versus tourniquet in robotic versus non-robotic assisted TKA.

Most importantly, the authors used an estimation of blood loss rather than a direct quantitative measurement. However, there is no “gold standard” for measuring blood loss, and there is no agreed-upon protocol for direct measurements [18]. Therefore, the authors opted for a more efficient manner of determining blood loss using previously described formulae which have been used for blood loss estimation for many years. These formulae have been determined to slightly overestimate blood loss as outlined in “Determination of Perioperative Blood Loss: Accuracy or Approximation?” [14]. Thus, for safety reasons, the authors assumed if any errors in estimation occurred, they most likely erred on the side of over-estimation rather than neglecting potential blood loss and undershooting blood loss estimations. This does, however, limit the applicability of the authors’ findings.

## Conclusions

The authors have shown that tourniquet adds no additional benefit for reducing blood loss in addition to TXA, and TXA alone has no deleterious effect on mean hemoglobin or length of hospital stay. Although similar results have been outlined in current literature, this study is one of a few studies available in the United States supporting the efficacy of TXA alone in TKA vs. TXA used with a tourniquet by directly comparing the two. Future studies should evaluate the most effective dosing of TXA to decrease blood loss in tourniquet-less TKA while minimizing complications such as thromboembolism. Prospective studies with long-term follow-up should be done in order to validate the findings of this study.
